# A Continuous Object Boundary Detection and Tracking Scheme for Failure-Prone Sensor Networks

**DOI:** 10.3390/s17020361

**Published:** 2017-02-13

**Authors:** Sajida Imran, Young-Bae Ko

**Affiliations:** Department of Computer Engineering, Ajou University, Suwon 443749, Korea; sajida@ajou.ac.kr

**Keywords:** continuous object detection and tracking, node failure, wireless sensor network

## Abstract

In wireless sensor networks, detection and tracking of continuous natured objects is more challenging owing to their unique characteristics such as uneven expansion and contraction. A continuous object is usually spread over a large area, and, therefore, a substantial number of sensor nodes are needed to detect the object. Nodes communicate with each other as well as with the sink to exchange control messages and report their detection status. The sink performs computations on the received data to estimate the object boundary. For accurate boundary estimation, nodes at the phenomenon boundary need to be carefully selected. Failure of one or multiple boundary nodes (BNs) can significantly affect the object detection and boundary estimation accuracy at the sink. We develop an efficient failure-prone object detection approach that not only detects and recovers from BN failures but also reduces the number and size of transmissions without compromising the boundary estimation accuracy. The proposed approach utilizes the spatial and temporal features of sensor nodes to detect object BNs. A Voronoi diagram-based network clustering, and failure detection and recovery scheme is used to increase boundary estimation accuracy. Simulation results show the significance of our approach in terms of energy efficiency, communication overhead, and boundary accuracy.

## 1. Introduction

Continuous object tracking is a useful application area of sensor networks for detecting and monitoring of roaming paths of continuous natured objects like forest fire, oil spills, flow of volcanic disposals, and hazardous biochemical diffusions [1,2]. Nodes that detect phenomena need to send sensed information along with their IDs to the sink [3]. Post-processing of data is done at the sink to extract useful information from raw sensing data, e.g., to estimate the phenomenon shape, spreading rate, and capacity. A large number of sensor nodes can detect phenomena at any given time [4,5]. Allowing all sensed nodes to send their detection status to the sink is quite expensive in terms of traffic overload and number of communications performed. A more efficient way is to let the nodes, present at the phenomenon boundary (BNs), send their detection status to the sink, which processes these boundary data to extract useful information [4]. This can greatly save traffic and communication overhead. However, in such a case, the boundary data should be reliable enough to accurately estimate the phenomenon boundary. Any inconsistencies in boundary data can result in significant boundary estimation errors. Failure of one or multiple boundary nodes (BNs) can significantly affect the quality and reliability of boundary data received at the sink and, hence, may reduce boundary estimation accuracy. Therefore, it is critical to develop an efficient approach that accurately detects BNs as well as detects and recovers from BN failures.

In the methods used in [6,7], a few representative nodes (RNs) are selected among BNs to send boundary data to the sink. A backoff timer is set by every BN to become an RN. However, these methods focus on compressing the number of sensed nodes and the number and size of report messages. RNs periodically send a report message to the sink even if the change in phenomenon shape is negligibly small, hence wasting the sparse resources of sensor nodes. Additionally, these approaches work on the assumption that sensor nodes can never fail. However, in reality, the tiny sensor modules can fail at any time owing to a hostile environment [8,9]. None of the existing works has considered the failure effects on continuous object detection and tracking. Failure of one or multiple nodes can affect the selection rate of BNs as well as may leave boundary coverage holes, which leads to inaccurate boundary estimation at the sink. An adequate failure detection and recovery scheme can significantly improve boundary tracking accuracy in case of node failures. 

In this work, we aimed to efficiently detect the boundary of a continuous object in a failure-prone network by utilizing the spatial and temporal features of nodes. Nodes whose current detection status is different from the previous one, checks the status of their neighbors to identify whether they exist inside or outside of the phenomenon boundary. Nodes whose current detection status remains the same as the previous one do not need to perform any action. The proposed approach utilizes two-hop neighbor detection information to avoid considering the minimal changes in the phenomenon shape. Our approach carefully chooses BNs by assigning weights based on the BN selection criteria fulfillment rate. We achieve improved boundary accuracy by detecting and recovering from node failures of object BNs. Node failures are detected through the exchange of messages between one-hop active nodes. Failure recovery is performed by awaking a spare node in the Voronoi cell where failure of node was detected. We only focus on failure detection and recovery of BNs to improve boundary data quality, and hence boundary estimation accuracy. To achieve communication efficiency, few leader nodes (LNs) are selected among BNs to report boundary data to the sink. The sink performs piecewise interpolation on received data to estimate the shape of the phenomena.

**Figure 1:**
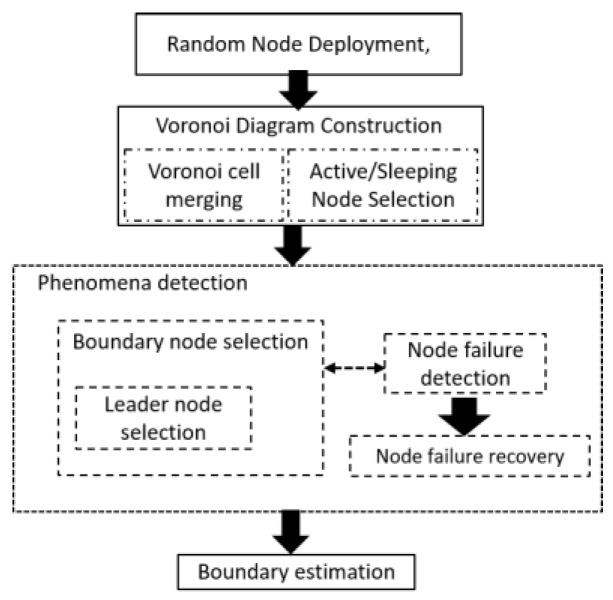
Overview of the proposed work.

Figure 1 shows an overview of the overall work done in this study. After the random node deployment, through the Voronoi diagram, nodes are partitioned into Voronoi cells. Each node in the network belongs to a Voronoi cell after Voronoi diagram construction is completed. Initially, the detection status of each node is 0. When phenomena emerge, the nodes that detect the phenomena change their status from undetected to detected, and exchange this change of detection status with one-hop neighbors. With this message exchange, some nodes are selected as BNs, and afterwards few LNs are selected among BNs. While the detection status is exchanged with one-hop neighbors, failure detection is performed using the same detection data. Finally, phenomenon boundary is estimated at sink using data sent by LNs.

We summarize the contributions of this work as follows. We used a Voronoi-based node clustering scheme that utilizes a sleep/wakeup approach to let only one node in a cell as active and put other nodes (redundant nodes) in the cell to sleep. The active nodes communicate with each other in a one-hop manner. We developed an optimized approach for BN selection, which takes into account two-hop neighbor information to ensure the reasonable emergence of the phenomena. LN selection is based on the number of one- and two-hop neighboring BNs and on the residual energy of the candidate, LN. We also developed a node failure detection and recovery algorithm to mitigate the effect of the failure of nodes present at the phenomenon boundary at any given time. The Voronoi-based clustering of nodes facilitate local detection and recovery from node failures. The proposed failure recovery scheme does not consider the movement of redundant nodes in place of the failed nodes. 

The rest of the paper is organized as follows. Section 2 and Section 3 describe the background and related works on the issues of continuous object detection and tracking, node failures, and the latter’s effect on the boundary detection accuracy. Section 4 presents models for networking and communications, and the proposed approach to detect and track phenomenon boundary, and detection and recovery of BN failures. Section 5 discusses the simulation results and analysis. Finally, Section 6 provides our conclusion.

## 2. Background

### 2.1. Continuous Object Tracking in Wireless Sensor Networks

Typically, object tracking consists of two operations: phenomenon monitoring and reporting of the monitored data back to the sink [10]. Nodes present at the phenomenon boundary are selected as the monitoring nodes to optimize the energy when transmitting the detection data to the sink. BN data are used at the sink to compute the phenomenon boundary at any given time. For applications in, e.g., forest fires, it is important to keep track of the emergence rate and the boundary of the fire expansion area. Continuous phenomena are usually spread over a wide area; thus, a large number of sensors are required for monitoring and tracking [11]. It is very inefficient to allow all the sensing nodes to forward their data to the sink. A more efficient way is to allow only the nodes present at the phenomenon boundary to send sensing information to the sink for boundary estimation. Detection of BNs for a continuously changing object is very challenging for a number of reasons: (1) The rapid changes in phenomenon shape also change the selected BNs. In one time slot, certain nodes may be at the phenomenon boundary, but in the next time slot, they may be inside the phenomena or no longer sensing the phenomena. (2) Phenomena evolve at an indeterminate pace, depending on the environment and the capacity of the phenomena. Sometimes, the change in phenomenon shape is so small that, when computed, it does not reflect any significant changes in the phenomenon boundary. (3) Transmission and communication are expensive tasks, considering the limited battery and computation resources of the sensing modules. Sending minor changes in the phenomenon shape wastes the sparse resources of the sensing modules and reduces the lifetime of the sensor network. (4) Nodes present at the phenomenon boundary can fail anytime and may leave coverage holes at certain boundary places, which can significantly affect the BN selection rate and boundary data quality. Any changes in BN selection will also reflect on the phenomenon boundary estimation accuracy. Therefore, careful selection of BNs is key to making an accurate boundary estimation.

### 2.2. Voronoi Diagram and Node Failure in Continuous Object Tracking

In wireless sensor networks (WSNs), nodes can fail for a number of reasons including, but not limited to, failure due to node damage, nodes running out of battery, and software failures. These failures can further be classified into single- and multiple-node failures [8,12]. In general, failure of nodes inside the network surrounded by other nodes can result in coverage holes and may partition the network. In continuous object tracking, failure of nodes present at or near the phenomenon boundary may affect the selection of BNs. Data from BNs are used by the sink for boundary estimation. The change in BN selection can decrease the estimated boundary accuracy. Therefore, it is important to detect and recover these failures. Different failure detection and recovery schemes are available in the literature [9,13,14,15,16]; however, there is no scheme available in the literature that considers and analyzes node failure in continuous object tracking.

A Voronoi diagram is a collection of nodes that partitions the space into polygons. To better understand the concept of the Voronoi diagram, let us assume that *X* is a metric space with distance function *d*. Let *K* be a set of indices and (*P_k_*)*_k_*_∈*K*_ be a tuple of nonempty subsets in space *X*. The Voronoi cell, or the Voronoi region, *R_k_*, associated with site *P_k_* is the set of all points in *X* whose distance to *P_k_* is not greater than their distance to other sites *P_j_*, where *j* is any index different from *k*. In other words, if (*d*(*x*, *A*) *= inf*{*d*(*x*, *a*)*|a*∈*A*} denotes the distance between point *x* and subset *A*, then *R_k_ =* {*x*∈*X|d*(*x*, *P_k_*) *≤ d*(*x*, *P_j_*) *for all j ≠ k*}*.* The sensor field is partitioned in such a manner that the nodes in each polygon should have a stronger connectivity with each other as compared to the neighboring nodes belonging to different polygons. To save energy and prolong network lifetime, we allow only one node in a cell to be active at a time. These nodes have one-hop connectivity with their active neighbors of surrounding cells. After the detection of node failure in a cell, any of the sleeping nodes is activated. The Voronoi cells are connected to each other and cover the entire network field. 

## 3. Related Work

### 3.1. Continuous Object Boundary Detection and Tracking

A multidimensional scaling-based boundary recognition (MDS-BR) algorithm was presented in [17]. The algorithm does not require the location information of the nodes; rather, it needs the two-hop neighbor information to construct the opening angles between nodes to decide on the BNs. An energy-efficient algorithm for autonomous real-time surveillance in sensor networks was proposed in [18]. The algorithm initiates and terminates tracks with lesser memory. The task of tracking is done hierarchically for real-time operation by forming a tracking group around a super node and later combining the tracks from different super nodes. To reduce the communication overhead, the algorithm first fuses the observations locally and then transmits them to a super node. In [13], static clusters are proactively made at the time of network deployment. In contrast, dynamic clusters are formed when sensors detect the appearance of some phenomena and send the detected information to the cluster head. However, the selection of dynamic clusters requires a significant number of communication attempts, especially when an object has a large radius and is monitored by many sensor nodes. Another energy-efficient boundary detection and tracking algorithm was proposed in [3]. The algorithm starts with the identification and selection of BNs. A node that receives a detection status different from its own status becomes a BN. Each BN maintains a boundary node array, where the neighboring detection status along with the node IDs is stored. The BN that receives the most distinct detection status compared to those of its neighboring BNs becomes an RN. The selected RNs send a BN array consisting of the IDs of the neighboring BNs to the sink. Although this approach reduces the energy consumption by optimizing the number and size of communication messages to the sink, the accuracy of the boundary detection and tracking of a continuous object is compromised. The COBOM algorithm proposed in [7] is similar to that in [14] in that it presents an energy-efficient algorithm that detects and tracks the boundary of a continuous object. Each boundary node maintains a BN array to keep the neighboring nodes’ IDs and their detection values. A few RNs are selected among BNs to send reports back to the sink. The sink estimates the boundary from the received data by exploiting the fact that, if the value ranges of two adjacent RNs are different, then a boundary will exist between these two nodes. COBOM provides an energy-efficient approach for tracking and detecting the boundary of a continuous object, but the accuracy of the boundary may be affected as COBOM’s report message does not include the neighboring BN’s location or ID. As a result, the sink cannot correctly estimate the boundary if two LNs are located far away. The idea of approximating the object boundary by a polygon is discussed in [19]. The interpolation points are placed by sensors having some sensed event information. The object boundary is estimated from uniformly placed interpolation points and from connected points. Every sensor node in a ring topology is responsible for distributing its local tangent and curvature information throughout the network. However, this approach uses a significant number of communication channels as the sensors in this approach are considered mobile and need to move to the interpolated points on the polygon after every update. In [5], to achieve energy efficiency in phenomenon tracking, the scheme restricts the active sensor nodes by selectively performing sleep/wakeup switching. A small set of collaborative nodes are responsible for detecting the boundary at any given time. The approaches in [4] address outer boundary issues, i.e., phenomenon expansion, shrinkage, and splitting as well as hole expansion, shrinkage, and splitting. Through local communications between edge nodes (of some phenomena or a hole), boundary nodes are identified. This process increases the energy efficiency of the object detection.

### 3.2. Detection and Recovery of Node Failure

In [8], the authors proposed a failing-node replacement algorithm. The replacement algorithm works by dividing the network into clusters. Each cluster head searches for redundant nodes inside the cluster. After detecting node failures through the detection message, the redundant nodes are moved to the locations of the failing nodes. The algorithm is not efficient because the cluster formation process puts an additional computational and communication burden. Moreover, redundant nodes have to move to the failing node position to restore the connectivity, which requires nodes to be energy rich and strong enough. These requirements are hard to meet in a WSN scenario. In [20], after it detects a node failure, if the failure does not partition the network, the topology remains the same. The approach considers recovering network partitions by placing substitute nodes at the positions of the failing nodes. However, as [20] only takes into account the situations having network partitions, the approach may leave coverage holes inside the network. Authors in [11] proposed graph theory-based heuristics to support K-connectivity. The process starts by forming a complete graph *G* with vertices set *V*. The edges are represented as set *E*, which connects the vertices. Each edge possesses some weight, which indicates the number of connected vertices. A convergence algorithm works well for WSN. In [21], the authors proposed a distributed algorithm for selecting the most critical node whose failure can slow down the convergence speed of an average consensus algorithm. The destruction rate is assessed by network algebraic connectivity. Three different algorithms are proposed to analyze the effect of node removal and corresponding estimation errors.

All these works consider the effect of sensor node failure in general. The proposed approach on the other hand considers node failures when detecting and tracking the continuous phenomena. The scheme works locally and detects a node failure through status message exchange between nodes. These status messages serve the dual purpose of exchanging status information and detection of node failures. The failure is recovered by activating any of the spare nodes present near the failed node and that have more common one-hop neighbors within a cell.

## 4. Proposed Scheme

In this section, a detailed description of the proposed approach is provided. First, different network, communication, and node deployment models for WSNs and their usage are explained. Then, the whole process of Voronoi diagram-based clustering, and active/sleeping node selection is explained. After that, object detection, BN selection, and LN selection is explained. At last, the proposed scheme for the detection and recovery of failed nodes and for the selection of active and redundant nodes is described.

### 4.1. Network Model

A network model describes the representation of nodes and how these nodes are connected to each other [22]. We represent the network model as *N* randomly deployed sensor nodes (*S =* {*s*_1_, *s*_2_, *s*_3_*…s_n_*}) and as vertices *v_i_* that belong to vertices set *V*, and the communication links between nodes as edges *e* that belong to a set of edges *E*. The number of edges *e* represents the connected one-hop neighbors of any node (node degree). The higher number of edges shows the strong connectivity of the nodes. We use a unit disk graph (UDG) as a communication model by considering a similar communication range of one unit between sensor nodes. Note that a UDG considers that all nodes have the same communication range that is normalized to one unit of length [23]. The way nodes are placed in the network has a significant effect on the overall network performance. There are many node deployment strategies available in the literature [24,25,26], which can be broadly classified into static and dynamic node deployment. In this paper, we assume the use of a static, yet random, node deployment strategy, as this work mainly focuses on applications where deterministic node deployment is difficult like in a forest fire.

### 4.2. Post-Deployment Network Clustering Using a Voronoi Diagram 

After the initial random deployment, nodes in the network exchange (via BOOTUP message) location information with their one-hop neighbors and the sink. The BOOTUP message contains the ID, location, and initial status (0 by default) of the sending node. After the completion of the message exchange, each node of the network has knowledge of its one-hop neighbors.

**Figure 2:**
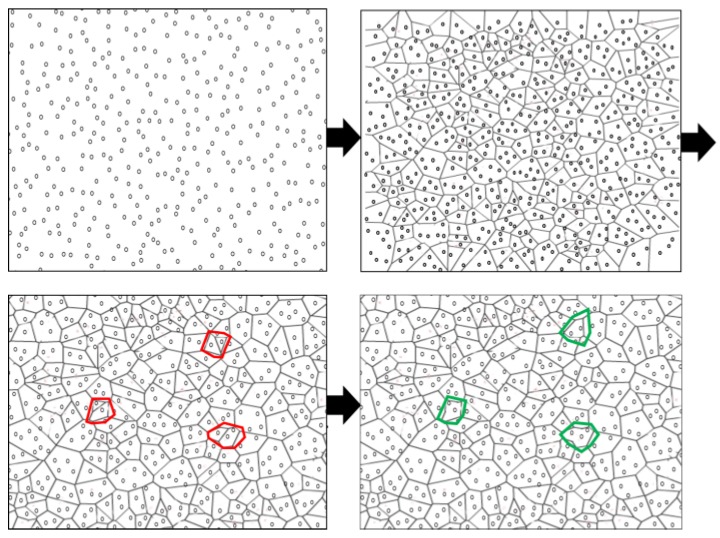
Voronoi diagram creation after node deployment. Upper left part of the figure shows initial random node deployment. Upper right part is the figure with Voronoi cell creation. Cells marked in red color on the bottom left shows an example of cells with very close nodes. These marked cells are merged into one cell. The bottom right section shows the Voronoi cells after merging. After that, only one active node per cell is selected and the rest of the nodes are put in sleep mode.

After BOOTUP message exchange completion, Voronoi diagram construction begins. Voronoi diagram-based network clustering conserves energy, prolongs network lifetime, and facilitates node failure detection and recovery. At first, the whole network field is divided into Voronoi cells (upper right part of Figure 2). Each Voronoi cell usually contains multiple sensor nodes. In dense networks where node deployment is random, it is likely that nodes may fall very close to each other such that most of their coverage area becomes overlapped. It is highly possible that sensing ranges of these closely residing nodes may cover more than one cell. To take this effect, after the initial development of the Voronoi diagram, Voronoi cells are merged into one if the nodes in these cells reach half of the sensing range of each other (bottom left of Figure 2). The merging of multiple cells optimizes the number of active nodes, as each cell has only one active node (bottom right of Figure 2). Nodes are clustered in such a way that active nodes can communicate with their one-hop neighbors. The active node selection is straightforward. Every node in the cell is capable of being active. The node having the least significant ID within the cell becomes an active node. The same process applies to all nodes in the Voronoi polygon. Each cell is assigned with a cell ID which is same as active node ID. Once selected, active node share its ID within its cell. It is important to share and maintain the active node’s ID within the cell. This cell ID is used to locally recover the failure effect by selecting another active node from the same cell of failing node. A Voronoi-based clustering, cell merging, and node activation is demonstrated in Figure 2.

### 4.3. Continuous Object Detection and Tracking

After deployment, BOOTUP message exchange, and Voronoi diagram construction, nodes are ready to detect and monitor the phenomena. To estimate the shape of the continuous object, nodes present at the boundary of the phenomena should be selected carefully. To preserve network resources, selected BNs should be small in number; however, at the same time, BNs should be in reasonable number to maintain boundary estimation accuracy. Continuous object boundary detection and tracking is a challenging task; any change in phenomenon shape is usually irregular owing to the nature of continuous objects and to different environmental factors such as air speed, temperature, and humidity. The proposed boundary detection and tracking scheme works in two steps: phenomenon boundary detection, BN selection, and LN selection. Before explaining the process, in Table 1 we describe some notations used in the proposed approach.

**Phenomenon detection and boundary node selection:** The detection process begins when nodes sense the existence of some phenomena. Usually, a substantial number of nodes are involved in the detection, depending on the phenomenon size. However, to save energy, only the nodes present at the phenomenon boundary (i.e., BNs) are considered to track the phenomenon shape. 

*Phenomenon detection:* We use the change in detection status of nodes and of their neighboring nodes for the detection and selection of BNs. Initially, the status of each node is set to 0. The sensing of phenomena is represented as status 1 or 0 otherwise. The change in detection status refers to either sensing or not sensing the phenomena at any given time. The change in value from 1 to 0 indicates that some phenomena were sensed in the previous time slot, but are not sensed in the current time slot. Moreover, the change in value from 0 to 1 indicates that some phenomena were not sensed in the previous time slot, but are sensed in the current time slot. 

**Figure 3:**
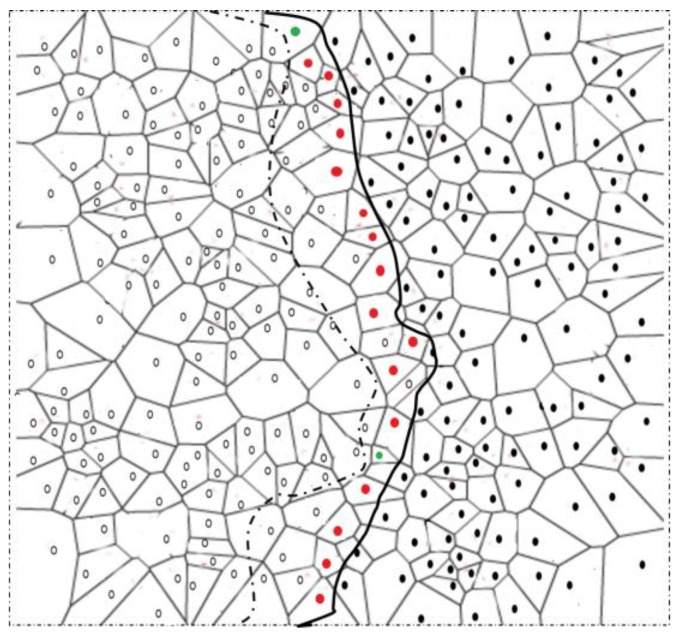
Boundary node selection. Nodes having a two-hop changed detection status become strong boundary nodes (BNs) (red dots), whereas nodes with a one-hop changed detection status become normal BNs (green dots). Nodes represented with hallow circles describe the detecting nodes, while nodes represented with solid black circles describe the non-detecting nodes at any given time.

**Figure 4:**
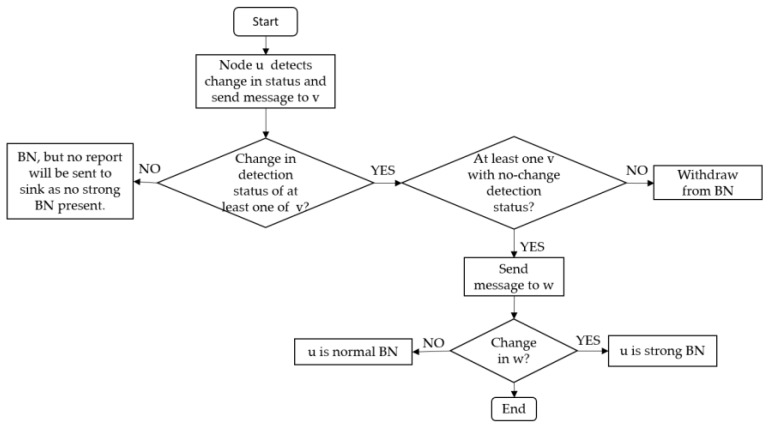
Flow diagram of the BN selection process.

*Boundary node selection:* A node with changed detection status sends its detection status to one-hop neighbors *v*. The neighbors *v* with changed detection status send the detection status to their one-hop neighbors *w*. If node *u* receives change in detection status of both *v* and *w*, it becomes strong BN. On the other hand, if *u* receives change in detection status of *v* and not of *w*, it becomes a normal BN. Note that for *u* to become either normal or strong BN, it should receive at least one unchanged detection status from *v*. This is to ensure that the selected BNs actually resides at the boundary of the phenomena. Algorithm 1 briefly explains the selection process of normal and strong BNs after node *u* detects a change in its detection status. Any change in the detection status of a two-hop neighbor confirms the capacity of the change in phenomenon shape. Algorithm 1 is graphically shown in Figure 3. Additionally, a detailed process of the phenomenon emergence, and strong and normal BN selection is explained through a flowchart in Figure 4.
**Algorithm 1** Algorithm for Boundary detection and BN selection**Input**: Node *u* detects a change in its detection status**Output:** Strong and normal BNs are selected**1.** Node *u* sends its detection status to one-hop neighbors *v*.**2.**
**if** there is a change in the detection status of *v*, **then**
**3.**  send a status message to one-hop neighbors *w*. **4.**  **if** the detection status of *w* is changed, **then****5.**  *v* send detection status of *w* to *u***6.**  **if** the detection status of both *v* and *w* is changed **then****7.**  *u* becomes strong BN**8.**  **else****9.**  *u* becomes normal BN**10.**  **else****11.**  a no-change message is sent back to node *u*.**12.**
**else****13.**  *u* withdraws to become a BN

**Figure 5:**
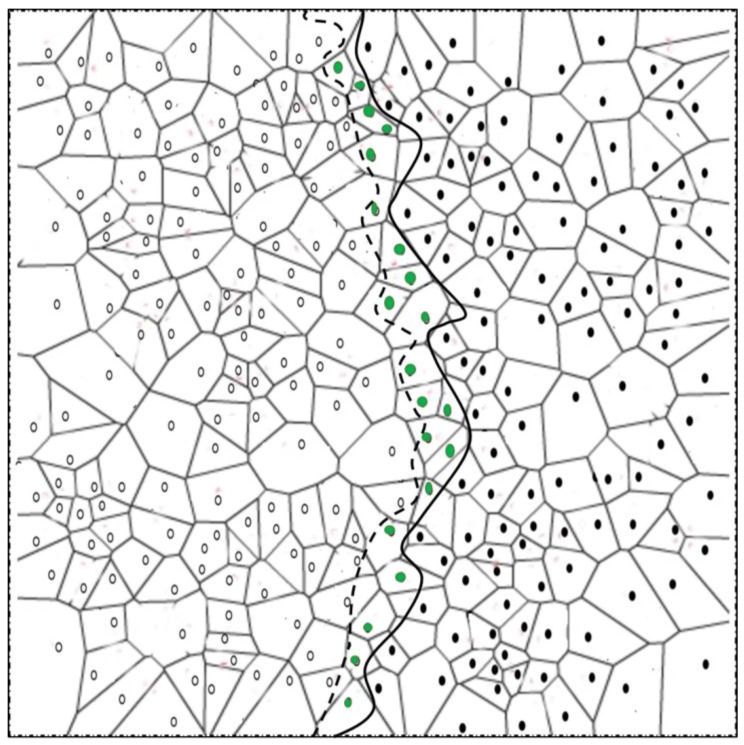
Change in phenomenon shape is negligible. Only normal BNs are developed. This trivial information is negligible and, thus, need not be sent to the sink to save network resources.

*Detecting negligible change in phenomenon shape:* Phenomenon detection data is reported to sink periodically. Depending on environmental factors like wind speed, phenomena may evolve negligibly in some time slots. Network resources can be saved by suppressing this trivial data to be reported to sink. Figure 5 shows an example in which the change in phenomena is so negligibly small that it covered only one-hop neighbors *v*. In the case, sending whole bunch of status report to the sink wastes sparse resources of WSN. Therefore, the proposed scheme sends the detection report only when at least one strong BN is present in a particular time slot. In Figure 5, as there is no strong BN present, this detection report is discarded. 

*Phenomenon expansion and contraction:* The change in detection status is noticed for both phenomenon expansion and contraction. For phenomenon expansion, BNs are selected from inside the phenomena, while, for contraction, BNs are selected from outside the phenomena. Owing to the irregular and large shape of the phenomena, it can expand in some parts, whereas it contracts in other parts simultaneously. Exactly the same procedure is applied to select BNs for both phenomenon expansion and contraction. There is no need to send the full detection information of the previous and new status together to the sink. It is clear that the sink can interpret the status message from a single value, i.e., if it gets a 1, it is likely that the node did not previously sense the phenomena but now senses them, whereas if the sink gets a 0, it can interpret that the node previously sensed the phenomena but does not sense it in the current time.

**Leader Node Selection:** Boundary nodes send their detection information to the sink, which perform computations on these data to estimate the phenomenon boundary. However, it is inefficient if all the selected BNs send their sensed information to the sink. Therefore, a few LNs are selected among BNs that send boundary detection data on behalf of their own and neighboring BNs. LNs need to be selected in such a way as to cover all the BNs and minimize/eliminate the overlapping of BNs in LNs. Once the selection of BNs is completed, the LN selection process begins. In this work, BNs that have a higher residual energy and a higher number of neighboring BNs have more chances of becoming LNs. Existence of more BNs indicate the importance of that BN’s position, as it can carry boundary data of more nodes and hence can aid in improved boundary accuracy. LNs are selected among BNs; therefore, every BN is a candidate for an LN. To become LN, each BN sets a backoff timer as in Equation (1).

(1) $$\mathrm{Boff}\_\mathrm{Timer}=\frac{w_{1}BN_{1d}}{w_{2}BBN_{d}E_{rd}}$$

Here, $BN_{1d}$ represents the number of normal *BNs*, *BBN_d_* represents the strong *BNs*, *Erd* represents the residual energy of *BNs*, and *w_1_* and *w_2_* are the weight of the weak and normal *BNs,* respectively. This is to ensure that a BN gets more chances of becoming an LN if it has more energy and is a strong BN*.*

### 4.4. Failure Detection and Recovery

**Figure 6:**
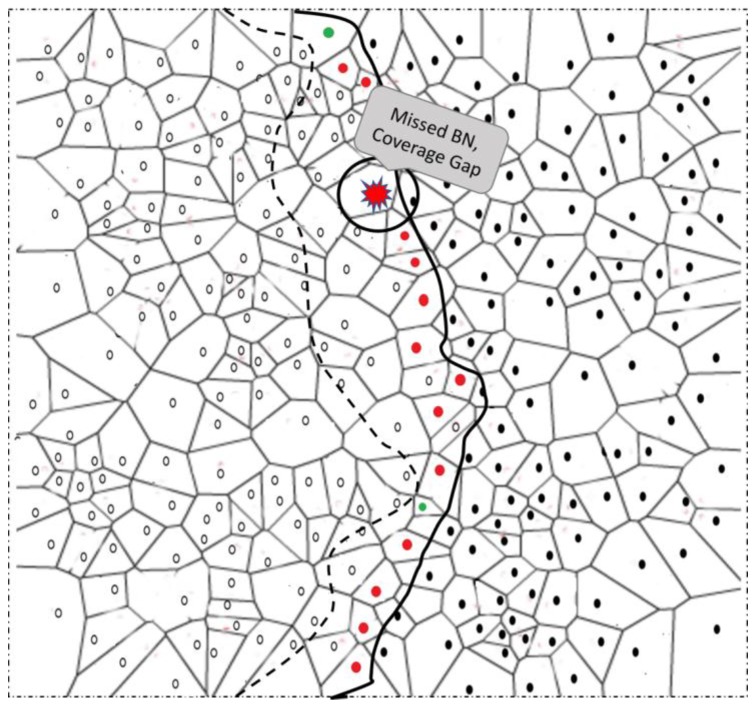
Example of a failing node and its effect on BN selection.

Sensor nodes in the network are subject to failure owing to a number of reasons: physical damage, malicious attack, and energy depletion. For accurately estimating the phenomenon boundary, it is important to detect and recover from BN failures. It is also important to find the critical nodes whose failure can affect the phenomenon detection and monitoring accuracy, more than that of any other nodes in the network. Figure 6 shows an example scenario where failure of a BN and its effect on the BN selection process are described. To consider these effects, we propose a node-failure detection and recovery strategy. We emphasized the approach, which should be light enough to be implemented locally for faster effects. On the basis of the Voronoi diagram, the strategy selects redundant/spare nodes and puts them to sleep. The proposed approach does not need movement of nodes to fill the positions of failed nodes; rather, it makes use of the already deployed spare nodes for failure recovery. Sensor mobility is an expensive task considering the sensor module’s tiny structure and limited resources. Moreover, the terrain may not be suitable for sensor nodes to move to specified locations; computing the specific location itself is a computationally intensive task. In the following section, we briefly explain the node failure detection and recovery process.

**Figure 7:**
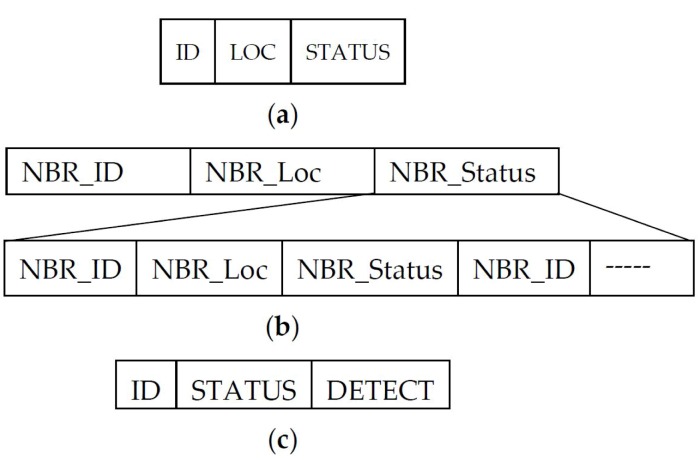
(**a**) BOOTUP message format. (**b**) Neighbor-table maintained by each node. (**c**) Detection status exchange message format. DETECT field is used to detect the node failure.

**Failure detection:** In the proposed approach, we are interested in detecting failed BNs, which affect the boundary detection accuracy. After the exchange of the initial ID and location information between one-hop neighbors (Figure 7a), each node in the network maintains a list of its neighbors (Figure 7b). In the BN selection process, when node *u* detects its changed detection status, it sends a detection message to its one-hop neighbors (Figure 7c). Node *u* expects a reply from all of its neighbors listed in its table of neighbors. Missing a reply from a neighboring node alarms the failure of that neighbor. The detecting node resends the status message with a tagged ID of the failed node. Resending of the status message confirms the failing of node if the node does not reply in the second attempt. Additionally, as this is a broadcast message, neighboring nodes learn about failed nodes and confirm the failure with their own experience. As opposed to previous approaches [8,15], our approach does not send a separate failure detection message to the neighbors; rather, a DETECT fields is included in the status message, which include the failure suspected node ID, to piggyback the detection status and its response between BNs. 

**Failure recovery:** After the detection of a failing node, recovery is performed locally. The proposed approach does not need the location of a failed node for recovery. Nodes that detect the failure, send an AWAKE message to the sleeping nodes in the respective cell. As cell ID is same as previous active node ID, the AWAKE message contains active node ID. Spare nodes only become active after receiving an AWAKE message. In case of more than one spare node in a cell, the node that receives more AWAKE messages will become an active node. A timer is set on the basis of the inverse relationship with the number of received AWAKE messages (Equation (2)).

(2) $$\mathrm{SPN}\_\mathrm{Time}=\frac{1}{No.\;of\;received\;AWAKE\;messages}$$

A spare node whose timer expired earlier than other spare nodes will notify itself as an active node. The remaining spare nodes go to sleep after receiving this message. Neighboring nodes delete the entry for failed nodes from their neighbor table. This process significantly increases the network lifetime and energy consumption as opposed to existing techniques where a redundant node is moved to the place of the failing node [8]. The failure detection and recovery process has a significant effect on the number of selected BNs and on the boundary estimation accuracy.

## 5. Performance Evaluation

### 5.1. Simulation Environment

Simulations were performed in a square area of 1000 m × 1000 m using a Java simulator. We used varying node densities from 500 to 2000 nodes. Phenomena were initiated at (0,0) of the bottom left corner of the sensor field and spread at a rate of 1 m per time slot. Data were collected after every 15 time slots to avoid any accidental errors. One hundred iterations were performed to analyze the results. The sink was placed at the extreme right upper part of the sensing field. To run the backoff timer, the weighting factor *w* was set to 0.3 for normal BN and default 1 for strong BN. To show the node failure effect, we randomly removed one of the BNs. The active mode and sleep mode power consumptions were 31 mW and 36 μW, respectively, whereas the transmitting and receiving power consumption rates were 75 mW and 63 mW, respectively [5]. We removed at most two BNs simultaneously to showcase node failures. This work did not consider any channel or propagation losses; rather, it focused on optimizing the number of BNs and LNs in the presence of failing nodes. The proposed scheme was compared with collaborative scheme [6] and COBOM [7]. 

### 5.2. Simulation Results

Sensor nodes periodically send phenomenon boundary detection data to the sink where it extracts boundary information from this sensing data and performs computations to estimate phenomenon boundary. The sensed boundary data should be collected accurately and delivered efficiently to make the boundary estimation reliable and cost effective at the same time.

**Figure 8:**
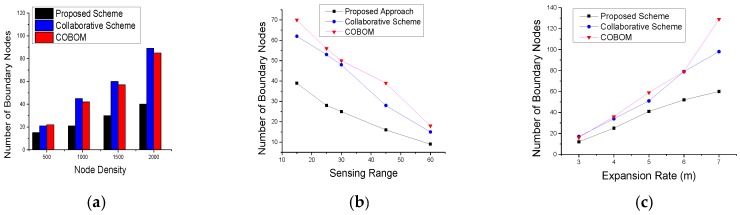
Number of boundary nodes with different (**a**) node densities; (**b**) sensing ranges; and (**c**) expansion rates.

The number of sensing nodes directly impacts the boundary data accuracy. However, the phenomenon detection and monitoring cost also increases as the number of nodes increases. The number of sensing nodes also depends on the pace of phenomenon emergence and the sensing range of the nodes. Figure 8 shows the number of BNs for different node densities, sensing ranges, and expansion rates. With the increase in node density, nodes become closer to each other and the phenomenon boundary is likely sensed by a great number of nodes. An efficient phenomenon detection approach should use an optimized number of sensing nodes while achieving reasonable boundary accuracy. In Figure 8a, the proposed scheme showed a significant reduction in BNs as compared to the other approaches. This is due to the consideration of a changed detection status of two-hop neighbors becoming BNs, and results in limiting the number of BN candidates. A collaborative scheme [6] showed the poorest results among the three comparative approaches. The collaborative scheme differentiates between inner and outer boundaries, which may provoke the selection of more BNs in the case of the presence of both types of boundaries.

The sensing range of a sensor node defines how far a node can sense an object. In phenomenon sensing, it affects the number of selected BNs as more nodes will share the same detection status which deviates the BN selection frequency. Figure 8b shows the result of the experiment with different sensor ranges and how it affects the number of selected BNs. In the proposed approach, the effect of the sensing range on BNs was significant as the nodes were placed far away from each other. To become a strong BN, the nodes have to detect phenomenon changes at the farthest location. This restriction limits the BN selection. In the collaborative scheme and COBOM, the BNs were selected on the basis of the changed detection status of one-hop neighbors only. Figure 8c shows the phenomenon expansion rate and its effect on the number of selected BNs. With an expansion rate of 3 m, the proposed scheme had the lowest number of selected BNs. COBOM [7] showed a significant increase in the number of BNs as the phenomenon expansion rate increased. The collaborative scheme’s increase in the number of BNs showed some stability when the phenomenon expansion rate was very high, this is because, sometimes change in detection status of inner and outer boundaries is same. 

**Figure 9:**
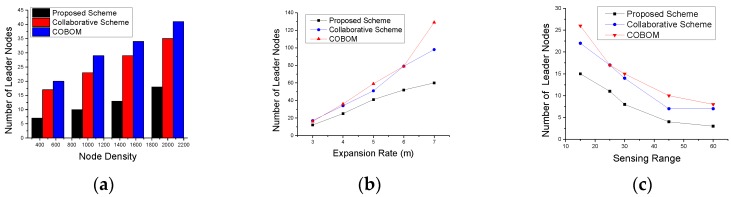
Number of leader nodes with different (**a**) node densities; (**b**) expansion rates; and (**c**) sensing ranges.

The effect of node density, the phenomenon expansion rate, and the sensing range also affect the number and frequency of selected LNs. With the increase in node density (Figure 9a), more BNs were selected that needed more LNs to carry their data to the sink. However, restricting the BN selection criteria in the proposed approach resulted in optimized LNs. Figure 9b shows the number of LNs with increasing expansion rate. We observed a decrease in LNs with the increase in the sensing range, as shown in Figure 9c. The proposed approach showed the lowest number of selected LNs for all sensing ranges, as compared to the other approaches. 

**Figure 10:**
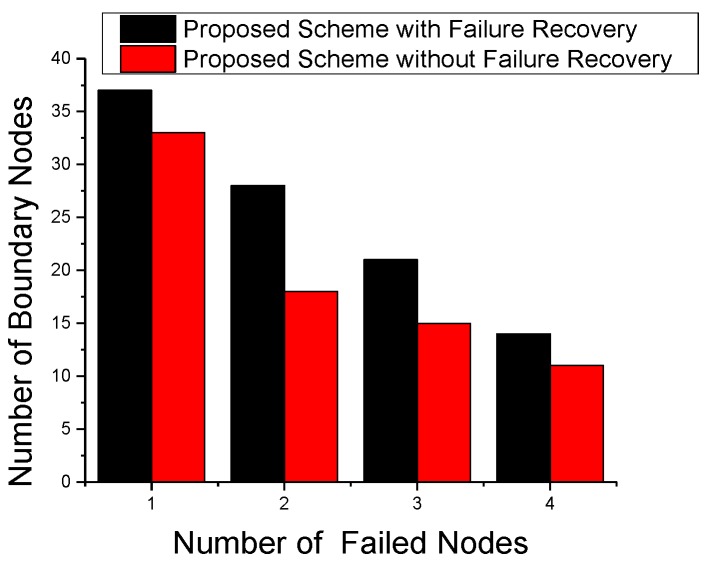
Effect of node failures on boundary nodes.

To analyze the effect of failing nodes on the number of selected BNs, we performed a node failure experiment. Figure 10 shows the number of selected BNs with node failure effects. We use maximum four failed nodes for this experiment. For the rest of failing nodes experiment, we use one and two failed nodes. The significance of failure effects depends on the position of the failing node at the time of BN calculation. With the increase in failing nodes, we see a decrease in the number of selected BNs as nodes with a changed detection status might not fulfill the criteria to become a BN. It is clear from Figure 10 that a good failure recovery strategy can significantly affect the object tracking performance in terms of BN selection.

To further analyze the effects of node failures on boundary tracking, we checked the boundary accuracy calculated by the sink using the data sent by the LNs. As phenomenon boundary detection data is sent by LNs that are selected among BNs, it is important to carefully select BNs and LNs. Moreover, BNs are selected based on the changed detection status of neighboring nodes. Failure of one or multiple neighboring nodes affect the number and frequency of BN selection and hence affects the novelty of boundary data received at the sink for boundary estimation. 

**Figure 11:**
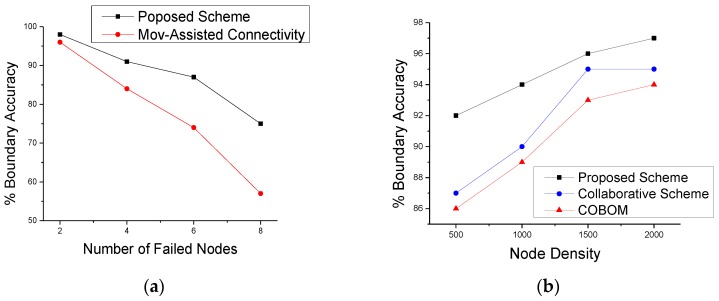
Boundary accuracy against different (**a**) numbers of node failures and (**b**) network densities.

**Figure 12:**
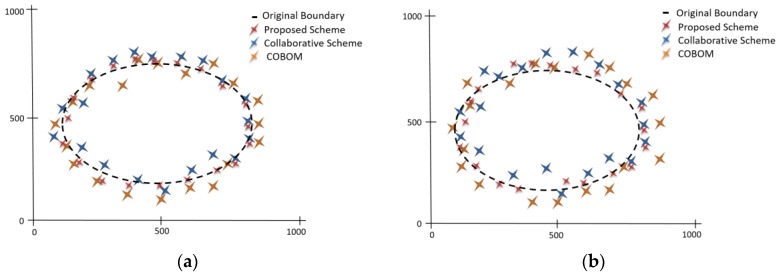
Phenomenon shape estimation considering a (**a**) single failed node and (**b**) two failed nodes.

For the boundary reconstruction at the sink, we used linear and polynomial interpolation methods [6]. This method takes LN positions as discrete points and constructs new points in between LNs as new boundary points. Figure 11a shows estimated boundary accuracy with varying number of node failures. The proposed scheme with failure recovery capability was compared with an existing scheme movement-assisted connectivity [16], which also considers node failure while detecting object boundaries. It is clear from the figure that the estimated boundary accuracy of the proposed scheme was significantly improved, compared to the scheme in the movement-assisted connectivity scheme. The proposed failure recovery scheme does not need the movement of sensor nodes to replace the failed nodes. The proposed scheme works locally to recover from node failure. Figure 11b shows the effect of different network densities on boundary accuracy. It is clear from the figure that the proposed approach showed an increase in boundary accuracy when the network density increased. We compared the estimated boundary accuracy of the proposed scheme with the collaborative scheme and COBOM. To do so, an eclipse was set at the middle of the network field. Actual boundary data represent LNs positions. We used piecewise interpolation to construct the phenomenon boundary. The boundary accuracy refers to the LN positions on the eclipse. Figure 12a shows the estimated boundary considering a single node failure, whereas Figure 12b shows the estimated boundary considering two failed nodes at the boundary. The proposed node failure and recovery scheme managed to cope with the effects of node failure and estimated the phenomenon boundary accurately. On the other hand, the comparative approaches failed to estimate the boundary accurately, as there was no mechanism that could be applied to detect and recover from node failures. 

**Figure 13:**
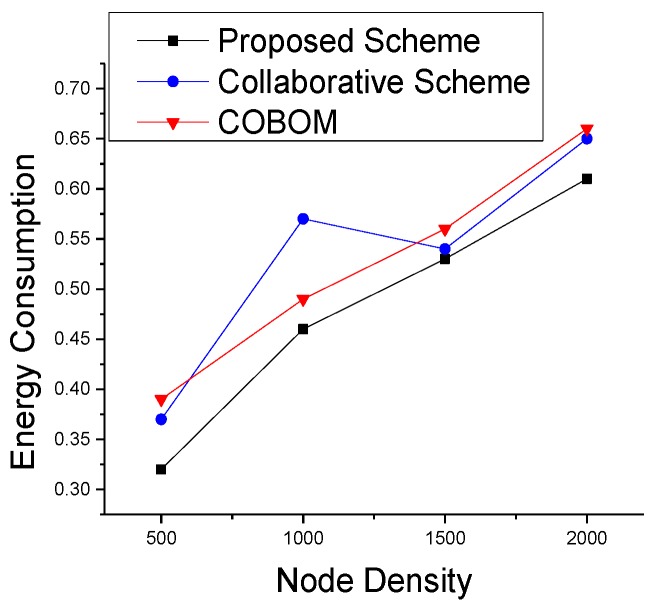
Normalized energy consumption with different node densities.

A novel boundary detection and tracking approach should accurately detect the phenomenon boundary using minimal network resources. Energy is the most important resource of a sensing module to keep on working. In Figure 13, we compared different approaches of analyzing the energy consumption in the phenomenon detection and tracking process. The active mode and sleep mode power consumption rates are set to 31 mW and 36 μW, respectively, whereas the transmitting and receiving power consumption rates are 75 mW and 63 mW, respectively. The energy consumption varied with different node densities. The proposed approach showed a smooth variation with the increase in node density. COBOM showed higher energy consumption with increasing densities because of the heavy control messaging for boundary detection and the exchanging of the boundary array. 

**Figure 14:**
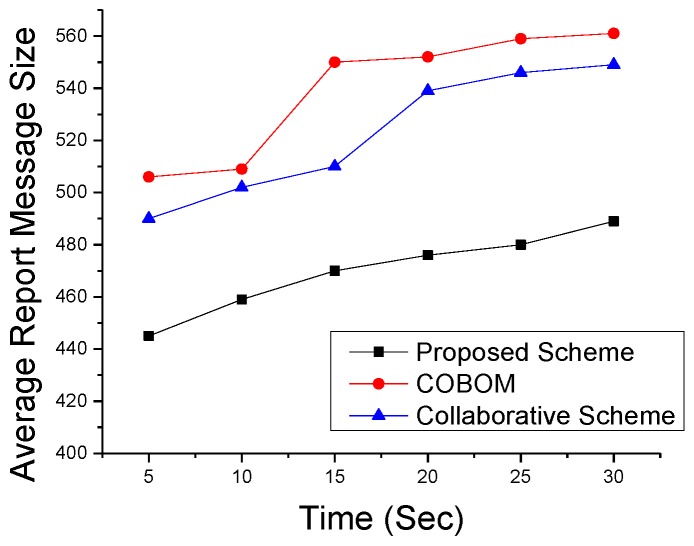
Averaged report message sizes at different times.

**Figure 15:**
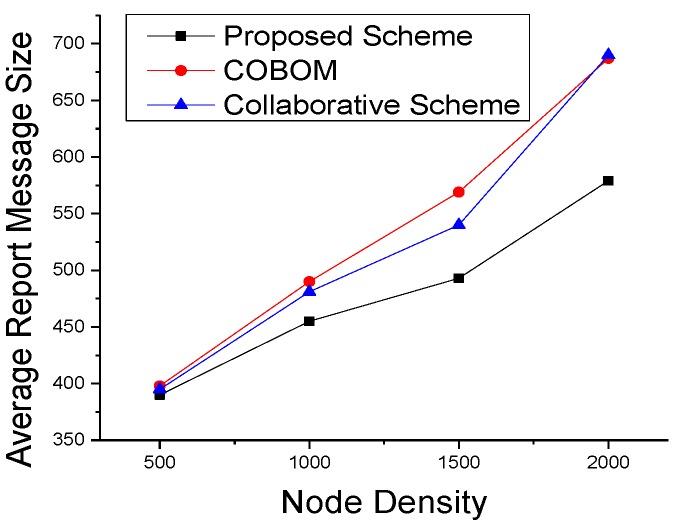
Averaged report message sizes with different node densities.

We also performed simulations to see the effect on data optimization and report the message size at different times in Figure 14. Data were averaged after each data collection interval. The figure shows a clear decrease in the proposed scheme’s data size, as compared to the other two boundary nodes, which may fall in the communication range of more than one LN. An LN forwards the data of its neighboring boundary nodes. As shown in Figure 15, the difference in report message size became high in the comparative approaches. The collaborative scheme’s report message size was optimized as compared to that of COBOM because it sends only the neighboring BN IDs in its report message. However, the collaborative scheme’s report message size increased at the fastest pace with the increase in node density.

## 6. Conclusions

In this paper, we presented a node failure-prone collaborative scheme for the detection and tracking of the boundary of a continuous object in WSNs. The scheme effectively detects and monitors the boundary of a phenomenon by exploiting the fact that a very small change in the shape of the phenomenon can be neglected to report to the sink, in order to conserve the sensor module’s limited resources. The optimization is achieved by restricting BN selection criteria based on one- and two-hop neighbors’ changed detection status and assigning weights to each BN accordingly. The proposed approach detects BN failures, which can cause inconsistencies in the boundary data at the sink and may lead to inaccurate boundary estimation. A failure recovery scheme is proposed to eliminate BN failures. The proposed scheme significantly reduces the report data size, as well as the number of communications performed, by suppressing trivial boundary detection data, and increases boundary estimation accuracy by detecting and recovering BN failures.

## Figures and Tables

**Table 1:** Frequently used notations with explanation.

Notation	Explanation
Boundary node (BN)	A node that receives at least one changed and one unchanged detection status of its one-hop neighbors.
Strong boundary node (SBN)	A BN that receives a detection status from its two-hop neighbors.
Leader node (LN)	A node that is selected among its one-hop neighbor BNs and sends its collected data from these nodes to the sink.
Node *u*	A node with a changed detection status.
Node *v*	A one-hop neighbor of node *u.*
Node *w*	A two-hop neighbor of node *u*.
